# Pathogenesis of *Helicobacter pylori*-Related Gastroduodenal Diseases from Molecular Epidemiological Studies

**DOI:** 10.1155/2012/371503

**Published:** 2012-07-05

**Authors:** Yoshio Yamaoka

**Affiliations:** ^1^Department of Environmental and Preventive Medicine, Oita University Faculty of Medicine, 1-1 Idaigaoka, Hasama-machi, Yufu-City, Oita 879-5593, Japan; ^2^Department of Medicine-Gastroenterology, Baylor College of Medicine and Michael E. Debakey Veterans Affairs Medical Center, 2002 Holcombe Boulevard, Houston, TX 77030, USA

## Abstract

*Helicobacter pylori* is a major human pathogen that infects the stomach and produces inflammation that is responsible for various gastroduodenal diseases. Despite the high prevalence of *H. pylori* infections in Africa and South Asia, the incidence of gastric cancer in these areas is much lower than in other countries. The incidence of gastric cancer also tends to decrease from north to south in East Asia. Data from molecular epidemiological studies show that this variation in different geographic areas could be explained in part by different types of *H. pylori* virulence factors, especially CagA, VacA, and OipA. *H. pylori* infection is thought to be involved in both gastric cancer and duodenal ulcer, which are at opposite ends of the disease spectrum. This discrepancy can also be explained in part by another *H. pylori* factor, DupA, as well as by CagA typing (East Asian type versus Western type). *H. pylori* has a genome of approximately 1,600 genes; therefore, there might be other novel virulence factors. Because genome wide analyses using whole-genome sequencing technology give a broad view of the genome of *H. pylori*, we hope that next-generation sequencers will enable us to efficiently investigate novel virulence factors.

## 1. Introduction

*Helicobacter pylori* is a gram-negative spiral bacterium whose ecological niche is the human stomach. It is a major human pathogen that infects the stomach and produces inflammation that is responsible for diseases, such as duodenal ulcer, gastric ulcer, gastric cancer, and mucosa-associated lymphoid tissue lymphoma. Despite a general decline in the incidence of gastric cancer, it remains the fourth most common cancer and second leading cause of cancer-related deaths worldwide (http://globocan.iarc.fr/). Interestingly, despite the high prevalence of *H. pylori* infections in Africa and South Asia, the incidence of gastric cancer in these areas is much lower than in other countries; these phenomena are called African enigmas and Asian enigmas [1] (Table 1). Furthermore, the incidence of gastric cancer has a tendency to decrease from north to south in East Asia. The pathogenesis of the different clinical outcomes is multifactorial with environmental factors (mainly diet) often playing a dominant role and with an influence by host factors, especially those governing the severity of the immune response as well as the virulence of the infecting organism.

*H. pylori*, which is highly heterogeneous, has a genome of approximately 1,600 genes, the majority of which have been functionally characterized, and 5% to 10% appear to be *H. pylori* specific [2, 3]. Genes that are specifically thought to be associated with virulence include *vacA, cagA, oipA*, and *dupA*. This paper describes the current knowledge about the pathogenesis of *H. pylori*-related diseases from the aspect of the virulence factors of *H. pylori*.

## 2. VacA (Vacuolating Cytotoxin)

Virtually all *H. pylori* strains have a functional VacA, which encodes a vacuolating cytotoxin. In addition to vacuolation, *vacA* can induce multiple cellular activities, including membrane channel formation, cytochrome *c *release from mitochondria leading to apoptosis, and binding to cell-membrane receptors, which is followed by the initiation of a proinflammatory response [4–6]. In addition, VacA can specifically inhibit T-cell activation and proliferation [7–9]. 

Differences in *vacA *structure at the signal (s) region (s1 and s2) and the middle (m) region (m1 and m2) contribute to variations in the vacuolating activity of different *H. pylori* strains [10]. s1/m1 strains are the most cytotoxic, followed by s1/m2 strains. However, s2/m2 strains have no cytotoxic activity, and s2/m1 strains are rare [10]. Many studies in Western countries, including Latin America, the Middle East, and Africa, have shown that individuals who are infected with *vacA* s1 or m1 strains have an increased risk of peptic ulcers and/or gastric cancer compared to those infected with s2 or m2 strains [10–12]. In East Asia, most of the *H. pylori* strains possess the *vacA* s1 genotype; therefore, the type of s region is independent of clinical outcomes [13, 14]. In contrast, the m1 genotype is common in areas of Northeast Asia, such as Japan and South Korea, whereas the m2 genotype is predominant in areas of Southeast Asia, such as Taiwan and Vietnam [14, 15]. Because the incidence of gastric cancer is higher in the northern regions than in the southern regions of East Asia, the *vacA* m region may play a role in the regional differences in the disease pattern in East Asia. We recently reported that the *vacA* m1 genotype was more prevalent in Hanoi than in Ho Chi Minh City in Vietnam, and the incidence of gastric cancer was higher in Hanoi than in Ho Chi Minh City [16]. These findings support the possibility that the *vacA* m region is related to clinical outcomes in East Asia. 

Okinawa consists of several small islands (2,276 km^2^) in southwestern Japan. Although the prevalence of *H. pylori* in Okinawa is not different from other parts of Japan [8, 13], the incidence of gastric cancer in Okinawa (6.3 deaths/100,000 population) is the lowest in Japan (mean mortality rate of Japan, 11.8 deaths/100,000 population in 2009) (Center for Cancer Control and Information Services, National Cancer Center, Japan, (http://www.ncc.go.jp/)). Interestingly, most of the *H. pylori* strains possess the *vacA* s1/m1 genotype in the mainland of Japan (e.g., Kyoto) [14]. However, we recently reported that less than 70% of the strains possessed the s1/m1 genotype in Okinawa [17]. In that study, we evaluated 337 strains and found that the* vacA* s1/m2 genotype was significantly prevalent in strains derived from gastritis than those derived from gastric ulcers (17.3% versus 7.9%, resp.; *P* = 0.04). The prevalence of the* vacA* s2/m2 genotype was significantly higher in strains derived from gastritis than those derived from gastric ulcers, duodenal ulcers, and gastric cancer (22.4% versus 11.9%, 10.5%, and 4.2%, resp.; *P* = 0.04, 0.01, and 0.04, resp.). Therefore, even in East Asia in areas where there are many cases with non-s1/m1 strains, both the *vacA* s and m genotypes can be used as markers for *H. pylori*-related diseases. 

In 2007, a third disease-related region of *vacA*, which was named the intermediate (i) region, was identified between the s region and the m region [18]. All s1/m1 strains were classified as i1 type, and all s2/m2 strains were classified as i2 type. However, s1/m1 strains were classified as either i1 or i2 types and i1 strains were shown to be more pathogenic. In a recent study, a novel intermediate variant (i3) was identified. This variant was often found in Turkish strains (25.7%) [19]. In the original study [18], it was reported that the *vacA* i genotype was more effective in determining the risk of gastric cancer than in typing the s region or the m region in Iran. An additional study that was conducted by the same group showed that the *vacA* i genotype was related to the presence of peptic ulcers in Iraq and Italy [20, 21]. Interestingly, a recent study from Republic of South Korea showed that the polymorphisms at amino acid position 196 of *vacA*, which is located in the i region, were associated with severe outcomes [22]. However, in our study in East and Southeast Asia, there were no associations between the i region and diseases [23]. In a recent study from Portugal that examined patients with progression to more severe histological diagnoses after a mean of 12.8 years of follow-up, the *vacA* i genotype did not improve the prediction of progression given by the other *vacAloci*, as in s and m regions [24]. More recently, we identified a fourth disease-related region between the i region and the m region and named it the deletion (d) region [25]. The d region is divided into d1 without a deletion and d2 with a 69 to 81 bp deletion. Our study of Western strains showed that d1 was a risk factor for gastric mucosal atrophy. However, almost all East Asian strains were classified as s1/i1/d1. Although the roles of the i and d regions should be investigated in a future study, the genotypes of the s and the m regions seem to currently serve as good markers of clinical outcomes. 

## 3. CagA (Cytotoxin-Associated Gene A Product)

*cagA* is located at one end of the *cag* pathogenicity island (PAI), which is an approximately 40 kbp region that is thought to have been incorporated into the *H. pylori* genome by horizontal transfer from an unknown source [26]. The *cag* PAI encodes a type IV secretion system (T4SS), through which CagA is delivered into host cells [27]. CagA has been reported to interact with various target molecules in host cells, and the best studied is the cytoplasmic Src homology-2 domain of Src homology-2 phosphatase (SHP-2), which is known to have oncogenic activity [28]. An animal study that used Mongolian gerbils showed that gastric cancer developed in animals infected with wild-type *H. pylori*, whereas it did not in gerbils infected with isogenic *cagA* mutants [29, 30]. Another study showed that gastric cancer and other malignant neoplasms occurred in some transgenic mice with an artificially introduced CagA protein [31]. These results provide strong evidence for the role of CagA as a bacterium-derived oncoprotein. 

There are 2 types of clinical *H. pylori* isolates: *cagA* gene-positive strains and *cagA* gene-negative strains. Almost all *H. pylori* isolates from East Asia are *cagA* positive, whereas approximately 20% to 40% of isolates from Europe and Africa are *cagA* negative [14]. Therefore, the pathogenic differences in East Asia are difficult to explain only in terms of the presence or absence of *cagA* alone [13]. In Western countries, however, it has been reported that individuals infected with *cagA-*positive strains are at a higher risk for peptic ulcer and/or gastric cancer than those infected with *cagA-*negative strains [32, 33]. It is interesting to note that almost all *cagA*-positive strains are classified as the *vacA* s1 strain (either m1 or m2), whereas almost all *cagA*-negative strains are classified as the *vacA* s2/m2 strain [10]. 

More than 10 years ago, we first reported that *cagA* could be mainly classified into 2 types (East Asian type and Western type) according to the sequence located in the 3′ region of *cagA* [34, 35]. We initially classified the repeat regions into 2 types, the first repeat and the second repeat, and found that the sequence of the second-repeat region was considerably different between East Asian strains and Western strains [14, 34–36]. Each region contains the Glu-Pro-Ile-Tyr-Ala (EPIYA) motifs, which includes a tyrosine phosphorylation site. Recently, it has been more common to name the first-repeat regions as EPIYA-A and EPIYA-B segments and the second-repeat region in Western and East Asian strains as EPIYA-C and EPIYA-D segments, respectively [28]. Each CagA sequence was assigned a sequence type that consisted of the names of the EPIYA segments in its sequence (e.g., ABC, ABCC, ABD). 

In vitro experiments have shown that CagA with an EPIYA-D segment has a higher binding ability for SHP-2 than CagA with an EPIYA-C segment [28]. An animal study showed that malignant neoplasms occurred in some East Asian-type CagA-introduced transgenic mice, whereas the frequency of tumors was significantly lower in Western-type CagA-introduced transgenic mice [37]. In addition, molecular epidemiological studies from Thailand and South Korea showed that individuals infected with East Asian-type *cagA* strains have an increased risk of peptic ulcer or gastric cancer compared with those infected with Western-type *cagA* strains [22, 38]. We also recently reported that the different incidences of gastric cancer between Okinawa and mainland Japan might be explained by the high prevalence of Western-type *cagA* strains in Okinawa compared with other areas of Japan [17]. In our study from Okinawa, the East Asian-type *cagA* genotype was significantly more prevalent in strains derived from gastric ulcers (83.2%) and gastric cancer (87.5%) than those derived from gastritis (60.2%) (*P* < 0.001 and *P* = 0.01, resp.). The prevalence of the East Asian-type *cagA* genotype was also significantly higher in strains derived from gastric ulcers (83.2%) and gastric cancer (87.5%) than those derived from duodenal ulcer (64.0%) (*P* = 0.001 and 0.02, resp.). In contrast, there was no significant difference between the prevalence of East Asian-type *cagA* in duodenal ulcers and gastritis (64.0% versus 60.2%). *H. pylori* infection is thought to be involved in both gastric cancer and duodenal ulcers, which are at the opposite ends of the disease spectrum. According to our results, this discrepancy can be explained in part by the prevalence of East Asian-type *cagA, *which might be specifically related to the development of gastric cancer. Overall, both in vitro and in vivo (animal and human) data clearly show that East Asian-type CagA is more carcinogenic than Western-type CagA. 

However, it should be noted that the incidence of gastric cancer is high in some regions where Western-type CagA is predominant. For example, although Western-type CagA strains have been reported to account for the majority of *H. pylori* strains in Columbia [4, 39], the incidence of gastric cancer there is substantially high (Table 1). These facts cannot be explained by the concept of East Asian-type CagA versus Western-type CagA alone. We published the first report that suggested that the number of second-repeat regions is associated with gastric cancer both in East Asia (Japan) and in Western countries, including Colombia [34, 35]. Importantly, our study of 100 *H*.  *pylori* isolates that were derived from patients with simple gastritis (30 isolates were from Columbia and 70 were from the USA, where the incidence of gastric cancer is low (Age-Standardized Rate = 4.1)) showed that 57% of the isolates from Columbia had 2 EPIYA-C segments, whereas only 4% of the isolates from the USA had 2 EPIYA-C segments [15]. Several studies have confirmed that the incidence of gastric cancer is significantly higher in patients infected with strains with multiple EPIYA-C segments compared with those infected with a single segment in Western countries [34, 35, 40, 41]. In addition, a recent large-scale study showed that a higher number of EPIYA-C repeats was associated with gastric cancer and gastric precancerous lesions, as shown by histological gastric atrophy/metaplastic changes and decreased serum levels of pepsinogen I [42]. The prevalence of *H. pylori* infections is high in Africa, while gastric cancer is uncommon, which is known as the “African Enigma” [43]. However, the incidence of gastric cancer is extremely high in Mali, and the frequency of gastric cancer among women in Mali is higher than in Japan (Table 1). It will be interesting to investigate the *cagA* genotypes in Mali. Taken together, the number of EPIYA-C segments may explain to some extent the geographic difference in the incidence of gastric cancer in Western countries. Somewhat interestingly, although we first reported that the risk of gastric cancer development in the Japanese population increased when the number of second-repeat regions was 2 compared with 1, the structure of the second repeat was not DD, but B′D, in which the sequences of B′ were more similar to B than to D [44]. Recent in vitro data have shown that SHP2 binds EPIYA-B segments and C-terminal Src tyrosine kinase (Csk), which is another important molecule that is involved in intracellular signaling systems and prefers to bind EPIYA-A and EPIYA-B segments [45]. These results might indicate that each EPIYA segment plays a role in gastric pathogenesis, and a larger number of any type of EPIYA segments might be used as a marker for an increasing risk for gastric cancer. 

As one goes southward in East Asia, the incidence of gastric cancer becomes lower, and the incidence in Vietnam is half of that in South Korea (Table 1), although most Vietnamese strains (93%) have been reported to possess the East Asian-type CagA [16]. In addition, most of the strains in both Vietnam and South Korea have only 1 EPIYA-A, EPIYA-B, and EPIYA-D segments [44]. Recently, we reported that the structure of the East Asian-type *cagA* in Vietnamese strains was slightly different from that of strains from other East Asian countries [16]. Vietnamese strains have a unique 18 bp deletion that is located slightly upstream of the EPIYA-A segment, whereas the 39 bp deletion is common in East Asian strains, such as those in Japan and South Korea, and no depletion was identified in Western strains. Further research is necessary to determine whether these subtypes are involved in the pathogenesis of gastric cancer.

## 4. OipA (Outer Inflammatory Protein)

OipA, which is one of the outer membrane proteins, functions in adhesion [46]. Its functional status is regulated by slipped-strand mispairing that is based on the number of CT dinucleotide repeats in the 5′ region of the genes (switch “on” = functional and switch “off” = nonfunctional) [46]. OipA was initially identified as a proinflammatory response-inducing protein based on the fact that *oipA*-isogenic mutants reduced the induction of interluekin-8 (IL-8) from gastric epithelial cell lines [46]. A recent study revealed that OipA has a function of inducing inflammation and actin dynamics through the phosphorylation of multiple signaling pathways that usually interact with *cag *PAI (CagA)-related pathways [47–52]. 

We previously examined the expression status or presence of multiple virulence factors (*cag* PAI, *vacA*, *iceA*, *oipA*, and *babA*) in different clinical outcomes [33]. *H. pylori* isolates were obtained from 247 patients in the USA and Colombia. An independent univariate analysis showed that the *oipA* “on,” *cag* PAI-positive, *vacA *s1 genotype and the *babA-*positive type were all related to the risk of duodenal ulcer. However, a multiple logistic regression analysis showed that only the *oipA* “on” status was an independent determinant predictor of duodenal ulcer from gastritis (adjusted odds ratio (OR), 5.0; 95% confidence interval (CI) = 2.1–11.9). This finding was confirmed in a distinct study that used a nonoverlapping cohort of 200 patients that were examined by an immunoblot analysis for 4 outer-membrane proteins: OipA, BabA, BabB, and SabA [53]. A multiple logistic regression analysis showed that only the OipA-positive status was an independent determinant predictor of gastric cancer versus gastritis (OR, 4.8; 95% CI = 1.4–16.8) and duodenal ulcer versus gastritis (OR, 4.0; 95% CI = 1.6–10.2). In addition, a challenge of human volunteers with an *oipA* “on”/whole *cag *PAI-negative clinical isolate (Baylor strain 100 or ATCC BAA-945) that caused severe inflammation supports this notion [54]. In addition, an in vitro study showed that the *oipA* mutants did not induce gastric mucosal inflammation in mice that were infected for 12 weeks, whereas *cagE* mutants did induce mucosal inflammation, although the levels were milder than in the parental strains (*cagE* is an important component of *cag* PAI) [55].

The above findings suggest that the presence of OipA is a better marker of severe clinical outcomes than *cag* PAI. However, it is important to note that clinical isolates that contain the *cag* PAI typically have an *oipA* “on” status [33, 53, 56–58] despite the *oipA* gene being physically located approximately 100 kbp from the *cag* PAI on the *H. pylori* chromosome. *oipA* status is also linked to the *vacA* s region type, and it is further closely linked to the presence of the *babA* gene, which is another virulence factor that codes outer membrane proteins [59]. These linkages of the virulence factors may have a certain biological significance, and they may somehow interact with each other; therefore, it might be better to hypothesize that these factors interact synergistically with each other and induce serious diseases, rather than to discuss which of these factors is the most virulent [15]. It is interesting to note that most East Asian strains are classified as *oipA* status “on,” and the CT-repeat sequences in the signal region of *oipA* were half-collapsed (e.g., CTGCCTTTCT repeat sequence), suggesting that this may result from an intentional change in the status in the course of evolution of the bacteria in order to prevent the switch from being turned “off” easily [46]. 

## 5. DupA (Duodenal Ulcer Promoting)

In 2005, we described a novel virulence factor, duodenal ulcer promoting (*dupA*) gene, which was located in the plasticity region of the *H. pylori* genome [60]. DupA pathogenesis appears to involve the induction of IL-8 production in the antrum, leading to antrum-predominant gastritis, which is a well-recognized characteristic of duodenal ulcer. Additionally, it has been reported that *H. pylori* containing intact *dupA* induces the IL-12 production of monocytes [61]. 

As for the molecular epidemiological studies, our initial study of a total of 500 *H. pylori* isolates, including 160 from Japan, 175 from Korea, and 165 from Colombia, showed that the positive rate for *dupA* was high in patients with duodenal ulcer and low in patients with gastric cancer, regardless of the patients' nationality (42% versus 9% on average) [60]. However, several controversial results have been reported worldwide, and an association between the presence of *dupA* and gastroduodenal diseases has appeared in some populations but not in others [15, 62]. *dupA *is generally more prevalent in Western strains than in Asian strains. In a recent review, the worldwide prevalence of *dupA* in patients with gastritis was reported to be 44.8%, and this value differed significantly between nationalities/ethnicities; *H. pylori* isolates from South America were significantly more likely to possess *dupA *(79.21% (160/202)) than those from East Asian (36.62% (130/355)), Middle Eastern (40.21% (39/97)), or European (43.75% (42/96)) countries [63]. The association between *dupA* status and disease development is primarily observed in Asian countries, such as China, Korea, Iraq, and North India. Our meta-analysis showed that infection with *dupA*-positive *H. pylori* increased the duodenal ulcer risk (OR, 1.41; 95% CI = 1.12–1.76), particularly in Asian countries (OR, 1.57; 95% CI = 1.19–2.06), but not in Western countries (OR, 1.09; 95% CI = 0.73–1.62) [64]. In contrast to the linkage among CagA, VacA, and OipA, most studies showed that there were no relationships between the presence of *dupA* and the presence of CagA, VacA, or OipA [64]. 

There are several possible explanations why the importance of *dupA* in gastroduodenal diseases has been controversial among studies. First, the discrepancy could be related to the limitations of the techniques used for detecting the intact *dupA* gene. All previous studies evaluated the presence of *dupA* by polymerase chain reactions and dot blot/Southern hybridization, but DupA proteins were not detected by immunoblot. However, it is well known that there are many cases with frame-shift mutations in *dupA*. Strains with these mutated sequences are not able to produce intact DupA proteins. Intriguingly, the presence of *dupA* without a stop codon was more frequently observed in strains from patients with duodenal ulcer than in those from patients with gastritis or gastric cancer [65]. Hussein et al. recently classified a *dupA *allele with 1,884 bp as *dupA1 *and a truncated version with mutations as *dupA2* [61]. Secondly, recent full-sequenced data of *H. pylori* revealed that the length of *dupA* depends on the strains, and the length of the Shi470 and G27 strains has an approximately 600-bp longer open reading frame (approximately 2,500 bp) than that of strain J99, due to the additional 5′ region of *dupA.* This suggests that *dupA* has 2 genotypes according to the location of the signal sequence of the 5′ region (long-type and short type). However, no previous studies took the additional 5′ region into account. Our preliminary data from Okinawa, Japan showed that the long-type *dupA* and not the short type *dupA* was significantly associated with severe gastroduodenal diseases (unpublished observation). A lack of concern about the 5′ region of *dupA* might be one reason for the discrepancies in the previous results. Although it is unknown whether proteins from short type *dupA* could be produced and/or functional, these data suggest that only strains that possess the long-type *dupA* without frame-shift mutations could be functional. Further analyses of the *dupA* DNA sequence will be necessary to clarify the significance of intact *dupA*. Additionally, intact *dupA* should be detected by measuring DupA protein with immunoblotting techniques.

Finally, *dupA* is predicted to form a T4SS with *vir* genes around *dupA* (*dupA* cluster). Three gene clusters that code for T4SS have been recognized in *H. pylori*: a protein translocation system encoded by the *cag* PAI, a DNA-uptake system encoded by the ComB cluster, and an unknown cluster in the plasticity region [66]. *dupA* and *virB4*, which is one of the constituents of T4SS, are highly homologous. *dupA *and the adjacent 6 *vir* gene homologs (*virB8*, *B9*, *B10*, *B11*, *virD4*, and *D2*) in the plasticity region were predicted to form the third T4SS [15]. We recently investigated the prevalence of *dupA* and *vir* gene homologs and the associations between the status of *dupA *clusters and clinical outcomes in the US population and found that the presence of a complete *dupA* cluster increases the duodenal ulcer risk compared with *H. pylori* infection with incomplete *dupA* clusters or without the *dupA* gene independent of the *cag* PAI status (adjusted OR, 2.13; 95% CI = 1.13–4.03) [66]. Therefore, although the causal relationship between the *dupA* cluster and duodenal ulcer development has not been proven, the presence of a complete *dupA* cluster and not *dupA* alone is associated with duodenal ulcer development. Overall, currently, the presence of a complete *dupA* cluster with intact *dupA *(long-type without frame-shift mutation) could be a good marker to predict the development of duodenal ulcer. Studies of the plasticity zone are only at the beginning and may be the most attractive area for future investigations.

## 6. Detection of Genomic Changes for Clinical Studies

The rapid advances in sequencing technology have enabled massive sequence comparisons. One of the prospective applications of the new technology to the study of *H. pylori* is the identification of novel virulence factors [67–69]. Whole-genome analyses are useful for the investigation of genetic factors that are related to differences in the virulence among strains. McNamara and El-Omar compared the genome sequences of an isolate that was obtained from a patient with gastric cancer (strain 98-10) and an isolate from a patient with gastric ulcer (strain B128) and determined strain-specific genes of strain 98-10 that were candidate genes associated with gastric cancer [70]. Kawai et al. investigated the evolution of East Asian strains using 20 whole genomes of Japanese, Korean, Amerindian, European, and West African strains [68]. A phylogenetic analysis revealed a greater divergence between the East Asian strains and the Western strains in genes related to virulence factors, especially those related to outer membrane proteins and lipopolysaccharide synthesis enzymes. Genomic changes during infection have also been studied. The whole-genome sequence of strain HPAG1 was determined with the whole-genome shotgun method, and the data obtained were used to design a custom microarray [71]. Genotyping of isolates that were obtained from patients with chronic atrophic gastritis revealed gained and lost genes during the progression of the disease, and whole-genome transcriptional profiling identified genes that were associated with the adaptation of *H. pylori* to chronic atrophic gastritis.

A chronological comparison of the whole genome was performed for 5 sets of *H. pylori* strains from Colombia with isolation intervals of 3 to 16 years using the 454 next-generation sequencing technology [72]. A comparison of the genomes revealed single-nucleotide polymorphisms and imported clusters that resulted from recombination, which is frequently found in members of the *hop* family. Data obtained with the massively parallel sequencing technology provide valuable information on candidates of new virulence factors.

## 7. Conclusions

It is obvious that the 4 virulence factors described in this paper are important. However, because *H. pylori* consists of approximately 1,600 genes, there remains the possibility that additional important pathogenic genes will be identified. The sequencing technology is still advancing. We believe that larger amounts of data will become available at lower costs in the near future, and other important novel virulence factors might be discovered. We must also note that the gastric cancer incidence has been changed remarkably with environmental factors, such as diet (e.g., salt intake) or immigration. Host factors (e.g., gene polymorphisms) and duration of the infection (e.g., early infection with duodenal ulcer and late infection with gastric cancer) should also be taken into account. These various factors are thought to interact in a complex manner with each other in the actual development of diseases. We hope that we will gradually understand the mechanisms underlying how *H. pylori* induces gastric inflammation and leads to severe gastroduodenal diseases, such as gastric cancer, by combining bacterial factors with other factors, such as environmental factors and host factors. 

## Figures and Tables

**Table 1:** Incidence of gastric cancer in 2008.

Geographic region	Country	Total		Male		Female	
		Total numbers	ASR	Total numbers	ASR	Total numbers	ASR
World total		989598	14.1	640556	19.8	349042	9.1
Asia		727500	18.6	484244	25.9	243256	11.7
East Asia		601314	30.0	408208	42.4	193106	18.3
West Asia		14879	9.4	9248	12.6	5631	6.7
Southeast Asia		43281	8.6	24926	10.9	18355	6.7
South-Central Asia		68037	5.3	41871	6.7	26166	3.9
Latin America and Caribbean		65360	11.7	39401	15.7	25959	8.4
South America		47244	12.4	29312	17.3	17932	8.4
Central America		14144	10.9	7671	12.7	6473	9.3
Caribbean		3972	8.5	2418	11.2	1554	6.1
Europe		146939	10.3	87548	14.7	59391	7.0
Central-East Europe		73940	14.7	43292	22.2	30648	9.7
South Europe		32873	10.1	19953	14.0	12920	6.8
West Europe		27457	6.5	16530	9.0	10927	4.4
North Europe		12669	6.2	7773	8.6	4896	4.2
Oceania		2728	5.5	1746	7.5	982	3.7
North America		24401	4.2	15051	5.8	9350	2.8
Africa		22659	4.0	12557	4.7	10102	3.3
(1) East Asia	South Korea	27098	41.4	18200	62.2	8898	24.6
(2) East Asia	Mongolia	603	34.0	390	48.2	213	22.3
(3) East Asia	Japan	102040	31.1	69561	46.8	32479	18.2
(4) East Asia	China	464439	29.9	315843	41.3	148596	18.5
(5) Central America	Guatemala	2332	26.6	1123	27.3	1209	25.9
(6) Central America	Honduras	1245	26.6	701	31.4	544	22.3
(7) South-Central Asia	Bhutan	114	24.2	76	31.6	38	16.2
(8) South America	Ecuador	3025	23.7	1667	28.0	1358	19.8
(9) South-Central Asia	Kyrgyzstan	964	23.2	619	34.2	345	14.5
(10) Central-East Europe	Belarus	3527	22.5	2023	34.2	1504	15.0
(11) Central America	Costa Rica	946	21.8	584	28.5	362	15.6
(12) South Europe	Albania	845	21.3	459	25.4	386	17.6
(13) South America	Peru	5215	21.2	2593	22.6	2622	20.0
(14) South-Central Asia	Kazakhstan	3329	20.6	1939	31.7	1390	13.7
(15) West Africa	Mali	1177	20.3	567	21.6	610	19.3
(16) South-Central Asia	Tajikistan	716	18.9	384	22.9	332	15.6
(17) Southeast Asia	Viet Nam	15068	18.9	8429	24.4	6639	14.6
(18) Caribbean	Jamaica	522	18.3	318	24.9	204	12.3
(19) South America	Chile	3762	17.9	2497	27.3	1265	10.3
(20) Central-East Europe	Russia	40615	17.5	22876	26.9	17739	11.7
(21) South America	Colombia	6638	17.4	3959	23.4	2679	12.5
(22) West Asia	Azerbaijan	1428	17.3	805	22.9	623	12.9
(23) Central-East Europe	Ukraine	13181	16.1	7902	25.2	5279	10.3
(24) South-Central Asia	Afghanistan	1716	15.8	1036	19.5	680	12.2
(25) South-Central Asia	Iran	8641	15.6	6188	21.9	2453	9.0
(26) South-Central Asia	Turkmenistan	532	15.4	310	21.2	222	10.9
(27) West Asia	Armenia	670	15.1	414	23.0	256	9.6
(28) South Europe	FYR Macedonia	468	15.1	315	22.7	153	8.6
(29) North Europe	Lithuania	916	15.0	532	23.0	384	10.0
(30) South Europe	Montenegro	149	15.0	85	19.2	64	11.5

ASR: age-standardized incidence rates per 100,000 population.

Data are obtained from GLOBOCAN databases, which provide access to the most recent estimates (for 2008) of the incidence of and mortality from 27 major cancers worldwide and is organized by the International Agency for Research on Cancer (IARC) (http://globocan.iarc.fr/).

In addition to the ASR for geographic regions, countries with ASRs that are equal or more than 15.0 for the total (male and female) with total number of gastric cancer more than 100 are listed.
